# The presence of tumour‐infiltrating neutrophils is an independent adverse prognostic feature in clear cell renal cell carcinoma

**DOI:** 10.1002/cjp2.204

**Published:** 2021-03-04

**Authors:** Basile Tessier‐Cloutier, David DW Twa, Mahsa Marzban, Jennifer Kalina, Hye‐Jung E Chun, Nils Pavey, Zaidi Tanweer, Ruth L Katz, Julian J Lum, Davide Salina

**Affiliations:** ^1^ Department of Pathology and Laboratory Medicine University of British Columbia Vancouver BC Canada; ^2^ Department of Pathology and Laboratory Medicine Vancouver General Hospital Vancouver BC Canada; ^3^ Faculty of Medicine University of British Columbia Vancouver BC Canada; ^4^ Life Science Institute University of British Columbia Vancouver BC Canada; ^5^ The Trev & Joyce Deeley Research Centre BC Cancer Victoria BC Canada; ^6^ Canada's Michael Smith Genome Sciences Centre BC Cancer Vancouver BC Canada; ^7^ Department of Pathology and Laboratory Medicine Royal Jubilee Hospital Victoria BC Canada; ^8^ Department of Pathology The University of Texas M. D. Anderson Cancer Center Houston TX USA; ^9^ Department of Biochemistry and Microbiology University of Victoria Victoria BC Canada

**Keywords:** clear cell renal cell carcinoma, tumour‐infiltrating neutrophils, immunohistochemistry, survival analysis, CIBERSORT

## Abstract

Tumour‐promoting inflammation is an emerging hallmark of cancer that is increasingly recognised as a therapeutic target. As a constituent measure of inflammation, tumour‐infiltrating neutrophils (TINs) have been associated with inferior prognosis in several cancers. We analysed clinically annotated cohorts of clear cell renal cell carcinoma (ccRCC) to assess the presence of neutrophils within the tumour microenvironment as a function of outcome. We centrally reviewed ccRCC surgical resection and fine‐needle aspiration (FNA) specimens, including primary and metastatic sites, from three centres. TINs were scored based on the presence of neutrophils in resection and FNA specimens by two pathologists. TIN count was correlated with tumour characteristics including stage, WHO/ISUP grade, and immunohistochemistry (IHC). In parallel, we performed CIBERSORT analysis of the tumour microenvironment in a cohort of 516 ccRCCs from The Cancer Genome Atlas (TCGA). We included 102 ccRCC cases comprising 65 resection specimens (37 primary and 28 metastatic resection specimens) and 37 FNAs from primary lesions. High TINs were significantly associated with worse overall survival (*p* = 0.009) independent of tumour grade and stage. In ccRCCs sampled via FNA, all cases with high TINs had distant metastasis, whereas they were seen in only 19% of cases with low TINs (*p* = 0.0003). IHC analysis showed loss of E‐cadherin in viable tumour cells in areas with high TINs, and neutrophil activation was associated with elastase and citrullinated histone H3 expression (cit‐H3). In the TCGA cohort, neutrophilic markers were also associated with worse survival (*p* < 0.0001). TINs are an independent predictor of worse prognosis in ccRCC, which have the potential to be assessed at the time of first biopsy or FNA. Neutrophils act directly on tumour tissue by releasing elastase, a factor that contributes to the breakdown of cell–cell adhesion and to facilitate tumour dissemination.

## Introduction

Accounting for up to 85% of all renal carcinomas, clear cell renal cell carcinoma (ccRCC) is by far the most common and aggressive renal malignancy [1]. Roughly a third of ccRCCs present with metastatic disease, and of those initially diagnosed with localised disease, 30% will recur and require systemic treatment [2]. Despite the development of targeted therapies, the 5‐year survival rates for renal carcinomas are still poor: 74% overall, 53% in patients with locoregional (stage III) disease, and 8% in patients with metastatic disease [3, 4]. ccRCCs are well known to have heterogeneous clinical outcomes, which makes patient stratification challenging, especially in lower‐stage disease. Molecular biomarkers have been disappointing, and the current reliable morphological features are limited to stage, grade, and the presence of necrosis [5, 6, 7, 8].

Tumour‐promoting inflammation is an emerging hallmark of cancer, as well as a potential treatment target in numerous tumour types [9, 10]. Lymphocytes and neutrophils are known to contribute to the microenvironment of ccRCC, and some studies have suggested they may also have prognostic value [11, 12, 13, 14]. ccRCCs are recognised to commonly elicit a strong immune response, and clinical trials have confirmed that a significant proportion of ccRCCs respond to immune checkpoint inhibitors [7]. The mechanisms by which neutrophils promote tumourigenesis are debated, but they are believed to be involved in tumour initiation through the generation of reactive oxygen species, reactive nitrogen species, and proteases in response to injury and tumour growth by expressing CXCR2 in response to interleukin (IL)‐17. Neutrophils also release elastase, cytokines, and other chemokines that promote angiogenesis via releasing vascular endothelial growth factor (VEGF), inhibiting both senescence and antitumour immunity, and stimulating renal carcinoma cell growth via upregulation of the c‐myc pathway [15, 16]. More recently, neutrophil extracellular traps (NETs), a novel cell death mechanism initially described to fight infectious agents by releasing web‐like structures composed of DNA, specifically modified citrullinated histones and cytoplasmic degradative enzymes (including elastase, matrix metalloprotease, and myeloperoxidase), have recently been shown to play a role in promoting tumour progression and tumour‐associated thrombosis in various cancers [17, 18, 19, 20, 21].

In view of the need for further stratification of ccRCC for clinical management, we analysed a clinically annotated cohort of ccRCCs with a known history of metastases. We set out to assess the role of neutrophils in ccRCC, looking at morphological features and neutrophil infiltration via immunohistochemistry (IHC) between primary and metastatic tumour tissue. Finally, we correlated the survival of a cohort of ccRCCs from The Cancer Genome Atlas (TCGA) with markers associated with the presence of neutrophils.

## Subjects and methods

### Patient samples

The cohort was enriched for metastatic ccRCC with tumour‐infiltrating neutrophils (TINs) with available tissue from both primary and metastatic resection specimens. Cases with resections were obtained from two centres: Vancouver General Hospital (BC, Canada) and Royal Jubilee Hospital (BC, Canada). Cytology specimens from fine‐needle aspirations (FNAs) of primary ccRCCs were obtained from a third centre: MD Anderson Cancer Center (TX, USA). The primary tumours were diagnosed between the years 1999 and 2016. Each case was centrally reviewed by two pathologists to confirm diagnosis and perform the pathology review. The project was approved by the University of British Columbia and Vancouver Island Health Authority Research Ethics Boards (H15‐01650 and H2015‐082).

### Pathology review

The diagnosis of ccRCC, grade, and stage were reviewed by two pathologists (BT‐C and DS). Formalin‐fixed paraffin‐embedded cases were scored based on the number of TINs and tumour‐infiltrating lymphocytes (TILs), separating cells surrounding necrosis and away from necrosis, as well as the presence of necrosis. In primary and metastatic resection specimens, we scored TINs and TILs using a scale of 0–3 (0 = 0, 1 = 1–10, 2 = 11–20, 3 = >20 TINs/TILs averaged over 10 high‐power fields [hpf]), after reviewing all slides from a given specimen and selecting the areas with the most inflammation. In FNA cytology specimens, TINs were scored according to the numbers of neutrophils infiltrating a minimum of 50 malignant cells/hpf using the same scale of 0–3. The cut‐off scores were defined arbitrarily to include the broad variation of inflammatory cells observed within our cohorts. We further grouped TINs as low TINs and high TINs to increase statistical power and reproducibility for our observations; details are included in the Statistics section below. The presence of ‘TINs around necrosis’ was defined by neutrophils next to viable tumour cells, within 0.5 mm of the necrotic tissue, whereas ‘TINs away from necrosis’ was defined by the presence of neutrophils next to viable tumour cells at least 5 mm away from the necrotic tissue.

### Immunohistochemistry

IHC was performed on a representative sample of cases with high and low TINs. Immunohistochemical staining followed previously published protocols [22]. For E‐cadherin and neutrophil elastase dual staining, slides were deparaffinised at 37 °C overnight. Slides were treated with peroxidase block for 5 min and Background Sniper (Biocare Medical, Pacheco, CA, USA) for 10 min. Neutrophil elastase staining was performed at 1:100 dilution (clone NP57; DAKO, Carpinteria, CA, USA) for 30 min followed by incubation with Mach2 Double Stain Ms‐AP polymer (Biocare Medical) for an additional 30 min and incubation with Ferangi blue chromogen (Biocare Medical) for 8 min. Antigen retrieval was performed with Diva decloaker reagents (Biocare Medical) in a Biocare decloaking chamber (110 °C for 15 min) and loaded on an Intellpath FLX autostainer (Biocare Medical). Peroxidase block and Background Sniper (Biocare Medical) steps were performed as described above, and staining for E‐cadherin (clone EP700Y; Epitomics, Burlingame, CA, USA) was performed at 1:500 dilution for 30 min followed by incubation with the Mach2 Rb‐HRP polymer (Biocare Medical) for an additional 30 min and incubation with DAB chromogen for 5 min. Haematoxylin (1:5 dilution) staining was conducted for 5 min. The E‐cadherin and PAX‐8 (clone CP379; Biocare Medical, 1:300 dilution for 30 min) staining was performed as described above with the Mach2 Rb‐HRP polymer. The anti‐histone H3 (citrulline R2+R8+R17, Clone ab5103; Abcam, Cambridge, UK, 1:100 dilution for 30 min) staining was performed as described above for elastase with DAB chromogen for 5 min. The denaturation step was performed using previously published protocols [23].

### Statistics

Chi‐squared and *t*‐tests were used for descriptive statistic comparisons. With overall survival (OS) and time to metastasis as the outcome variables, Kaplan–Meier survival curves were used to visualise differences in survival with respect to the presence of TINs, TILs, and necrosis. Analyses were performed separately for each cohort. In primary and metastatic resection specimens, a score of 0 was considered low, whereas scores of 1, 2, and 3 were considered high. In FNA cytology specimens, scores of 0 and 1 were considered low and scores of 2–3 high. Significance for Kaplan–Meier analysis was determined using the log‐rank test. For the TCGA samples, the Cox model was built for neutrophil enrichment, controlling for sex, age, and disease severity. RStudio (v1.0.153) was used to perform survival analysis using the survival package (v2.41‐3) in R (v3.3.3).

### CIBERSORT analysis of TCGA KIRC RNA‐seq data

For gene expression analyses of TCGA kidney renal clear cell carcinomas (KIRC), we obtained Fragments Per Kilobase of transcript per Million mapped reads (FPKM) values from the TCGA data portal (level 3 data available as of 29 May 2019), which represented normalised expression levels of 60 483 genes (based on EnsEMBL annotation) from 516 treatment‐naïve KIRC samples that underwent RNA‐seq.

To deconvolute gene expression signals originating from different immune cell types and to estimate the extent of immune cell presence within a tumour sample, we performed CIBERSORT analysis on the gene‐level FPKM data using the CIBERSORT R script (version 1.04) (University of Stanford, Stanford, CA, USA) [24] and applied 5000 permutations and the absolute signature score mode.

## Results

Our cohort included 102 ccRCC samples comprising 65 resection specimens (from 49 different patients) and 37 FNAs. The resection specimens were separated into two groups, primary (*n* = 37) and metastatic (*n* = 28) resections, 16 of which were matched. Patient and tumour characteristics are summarised in Table 1.

**Table 1:** Clinicopathological features of the ccRCC cohorts (*n* = 102).

Clinical/molecular features	Resection specimens, primaries (*n* = 37)	Resection specimens, metastases (*n* = 28)	FNA specimens, primaries (*n* = 37)
Sex (male)	25/37	25/28	–
Age (years)			
Range	33–82	33–82	–
Median	63	62	–
Tumour size (cm)			
Range	1.2–17	2.5–15.5	2–15
Median	7.5	9	6
Metastasis at diagnosis	N/A	N/A	22/32
Metastasis progression	21/37	28/28	25/37
Most common site of metastasis	Spine (*n* = 4)	Spine (*n* = 4)	–
WHO/ISUP grade			
Grade 1	6	3	4
Grade 2	13	6	16
Grade 3	8	12	18
Grade 4	9	5	4
Tumour stage			
pT1	15	8	8
pT2	2	1	4
pT3	19	18	15
pT4	1	0	1
Lymphovascular invasion	12/36	13/27	
Positive margins	6/37	3/28	N/A
Tumour necrosis	15/37	10/28	N/A
Inflamed necrosis (0–3)			
Score 0	0	1	N/A
Score 1	2	4	N/A
Score 2	4	2	N/A
Score 3	9	3	N/A
Neutrophils around necrosis (0–3)			
Score 0	1	3	N/A
Score 1	2	1	N/A
Score 2	3	2	N/A
Score 3	9	2	N/A
Neutrophils away from necrosis (0–3)			
Score 0	23	8	N/A
Score 1	3	11	N/A
Score 2	7	5	N/A
Score 3	4	4	N/A
Neutrophils (0–3)			
Score 0	N/A	N/A	4
Score 1	N/A	N/A	12
Score 2	N/A	N/A	3
Score 3	N/A	N/A	18
Intratumoural lymphocytes (0–3)			
Score 0	9	16	N/A
Score 1	11	5	N/A
Score 2	12	3	N/A
Score 3	5	4	N/A
Peritumoural lymphocytes (0–3)			
Score 0	1	3	N/A
Score 1	11	9	N/A
Score 2	22	9	N/A
Score 3	3	6	N/A

–, Not available; N/A, not applicable; WHO/ISUP, World Health Organization/International Society of Urological Pathology.

### Morphological review of the ccRCCs

**Figure 1:**
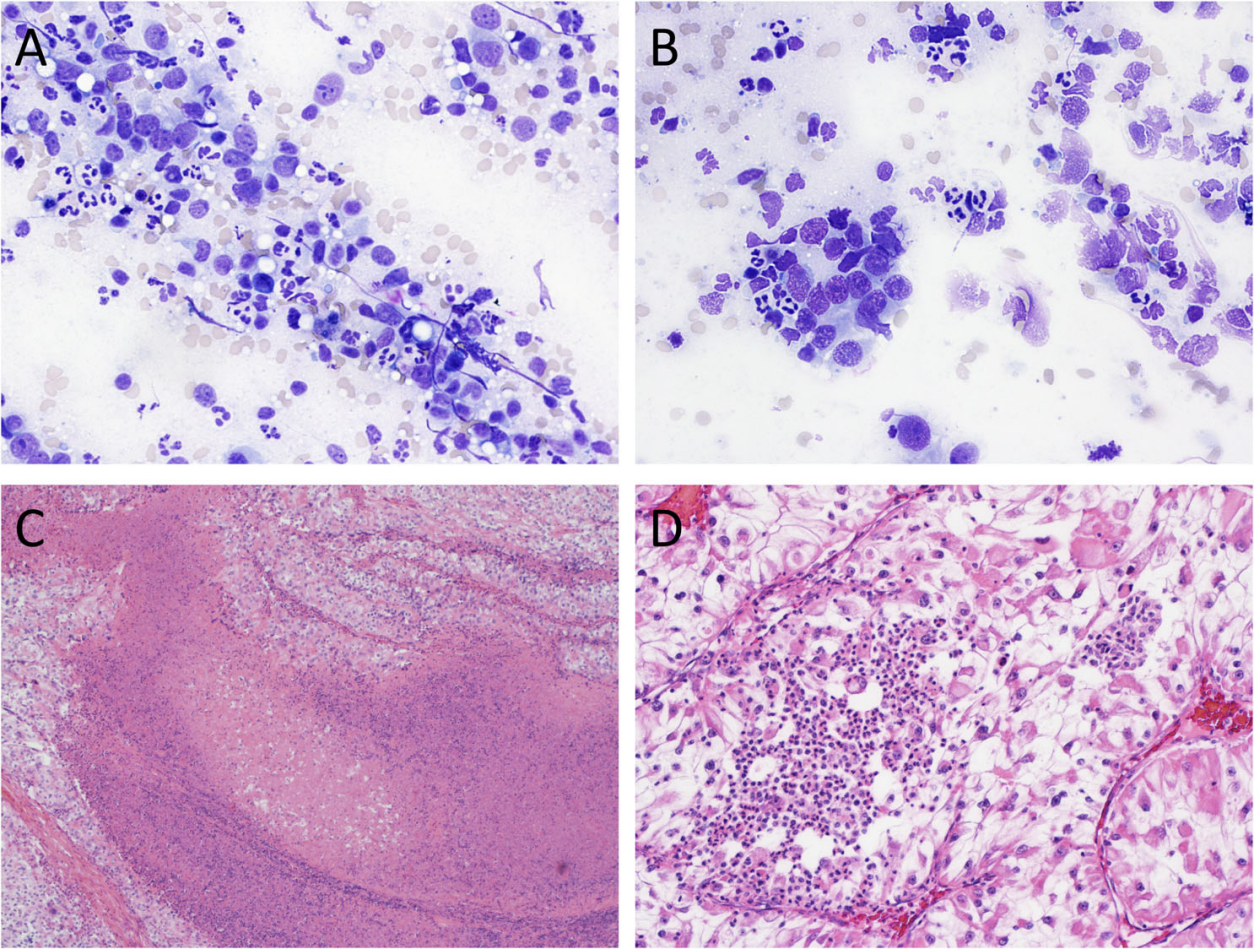
Images of different clear cell renal cell carcinomas showing high TINs in fine‐needle aspirate samples (A and B) and from a resection specimen (C and D).

At high power, neutrophils were tightly associated with malignant tumour cells and not dissociated in the peripheral blood elements; the latter was best seen on Diff‐Quik‐stained FNA samples (Figure 1A,B). In tissue sections, the tumours with high TINs and necrosis had extensive blue staining ‘nuclear dust’ from geographic tumour necrosis infiltrated with neutrophils (Figure 1C). The neutrophils within the necrotic areas can be easily identified with either myeloperoxidase or elastase staining. The presence of ‘TINs away from necrosis’ was defined by neutrophils at least 5 mm away from necrotic tissue. These usually appear as microabscesses or small collections of neutrophils without any geographic tumour necrosis (Figure 1D). Within the primary resection cohort (*n* = 37), 23 cases had low and 14 high TIN counts. The breakdown of the clinicopathological characteristics within the low and high TIN groups is presented in Table 2.

**Table 2:** Clinicopathological characterisation of the cohort separated by the presence of low (score 0) or high (score 1–3) TIN count.

Clinical/molecular features	Neutrophils away from necrosis	
	Low TINs (%)	High TINs (%)
Sex (male)	13/23 (57)	12/14 (86)
Age (mean in years)	61	61
Tumour size (mean in cm)	6	10
Metastasis at diagnosis	2/23 (9)	4/14 (29)
Grade (≥2)	16/22 (73)	13/14 (93)
Tumour stage (≥2)	10/23 (43)	11/13 (85)
LVI	5/23 (22)	7/14 (50)
Margins	4/23 (17)	2/14 (14)
Necrotic tissue	5/23 (22)	10/14 (71)
Inflamed necrosis	2/7 (29)	10/10 (100)
Intratumoural lymphocytes	7/23 (30)	10/14 (71)
Peritumoural lymphocytes	11/17 (65)	8/13 (62)

LVI, lymphovascular invasion.

### In primary and metastatic tumours, the presence of TINs was associated with worse outcome

**Figure 2:**
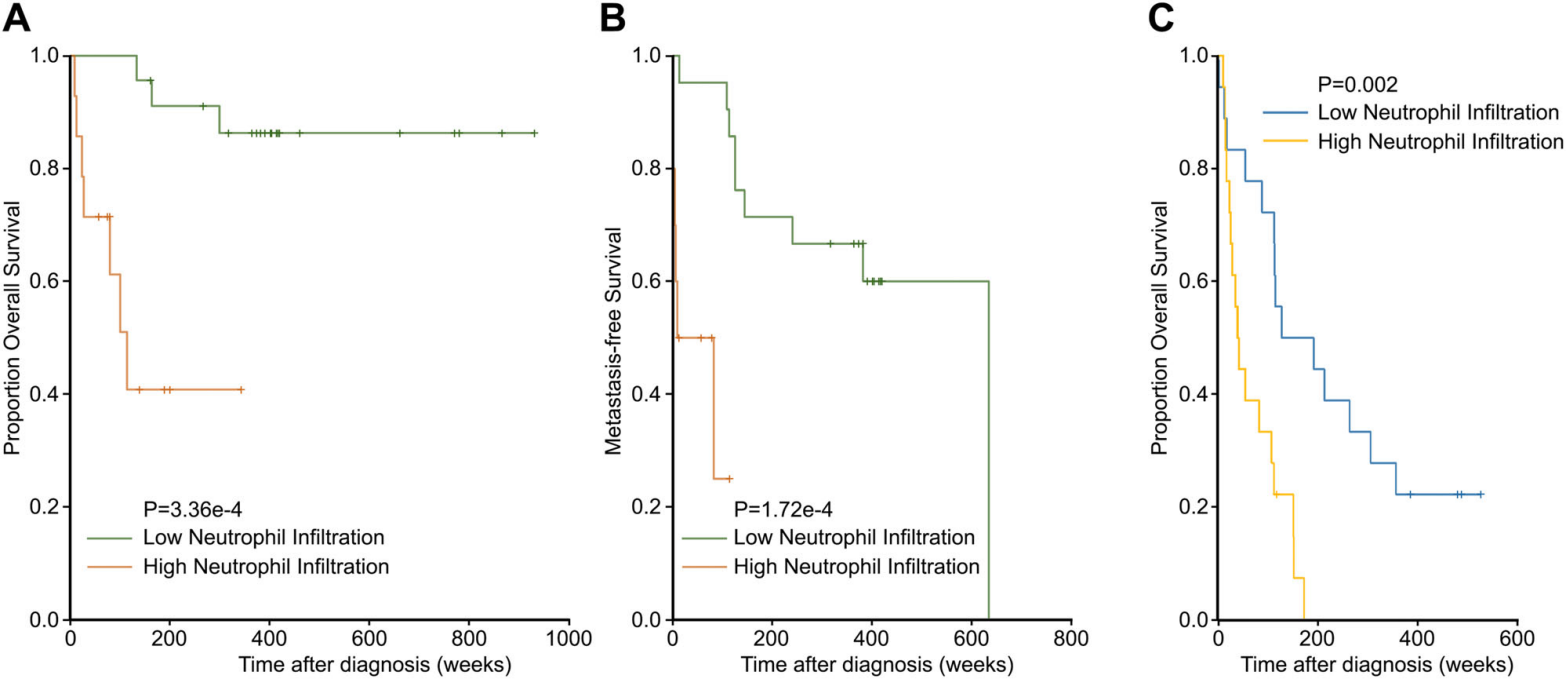
Kaplan–Meier analysis of low and high TIN count in association with (A) OS and (B) time to metastasis in the resection specimen cohort and (C) OS in the FNA sample cohort.

In resection specimens, the presence of high TINs (close and far from necrosis) was associated with worse outcome in both primary and metastatic tumours (*p* = 0.009) (Table 1). However, when in isolation, high TINs (scores 1–3) away from necrosis reached significance (*p* = 0.0003 for primaries and *p* = 0.02 for metastases), while high TINs surrounding necrosis did not (*p* = 0.07). The presence of necrosis was not significantly associated with outcome (*p* = 0.05). High TINs were associated with shorter OS (24.0 versus 103.4 average months OS, *p* = 0.0003) and a shorter time to metastasis (15.6 versus 67.2 average months to metastasis, *p* = 0.0002) (Figure 2). Analysis of other clinicopathological characteristic of the cohort showed that pathological stage, tumour grade, and size were also statistically significant (Table 3). In Cox proportional hazards regression analysis, TINs away from necrosis retained statistical significance (*p* = 0.03) (Table 4). When using Cox modelling to predict worse OS, larger tumour size and the presence of neutrophils away from necrosis were the two most important variables in primary tumours (*p* = 0.0002). In metastatic tumours, high stage and the presence of neutrophils away from necrosis were the best predictors of worse outcome (*p* = 0.004). In primary tumour resections, high TINs around (*p* = 0.003) or away (*p* = 0.0002) from necrosis were associated with a shorter time to metastasis compared to low TINs (score 0), whereas the presence of necrosis, again, was not a significant variable. When comparing the primary and matched metastasis in 16 patients, there was a trend towards more TINs and TILs in metastatic tissue when the primary tumour had high TINs and/or TILs (*p* = 0.07 and 0.1, respectively). The limited sample size in this case could explain the lack of statistical significance. Peripheral neutrophilia was excluded in all cases with TINs.

**Table 3:** Kaplan–Meier analyses of clinicopathologic features.

Clinical/molecular features	*P* statistic for primaries (*n* = 37)	*P* statistic for metastases (*n* = 28)	*P* statistic for FNAs (*n* = 37)
Sex	NS	NS	
Age	NS	NS	
Tumour size	0.003	0.03	0.006
Metastasis at diagnosis	NS	NS	0.01
Grade	0.03	NS	NS
Tumour stage	0.003	0.02	0.0006
LVI	NS	NS	
Margins	NS	NS	
Necrotic tissue	0.05	NS	
Inflamed necrosis	NS	NS	
Neutrophils around necrosis	0.07	NS	
Neutrophils away from necrosis	0.0003	0.02	
Presence of neutrophils	0.009	NS	0.00009
Intratumoural lymphocytes	NS	NS	
Peritumoural lymphocytes	NS	NS	

LVI, lymphovascular invasion; NS, not significant.

**Table 4:** Cox overall survival combining both primary and metastatic resection specimen samples (*n* = 65) (continuous independents).

Clinical/molecular features	*P* statistic for primaries (*n* = 37)	*P* statistic for metastases (*n* = 28)	*P* statistic for FNAs (*n* = 37)
Age	NS	NS	
Year of diagnosis	NS	NS	
Tumour size	0.0004	0.02	0.02
Grade	0.02	NS	NS
Tumour stage	0.004	0.02	0.0002
Inflamed necrosis	NS	NS	
Neutrophils around necrosis	NS	NS	
Neutrophils away from necrosis	0.03	0.03	
Presence of neutrophils	0.06	NS	0.001
Intratumoural lymphocytes	NS	NS	
Peritumoural lymphocytes	NS	NS	
Cox modelling (tumour size and neutrophils away from necrosis)	0.0002	NA	NA
Cox modelling (tumour stage and neutrophils away from necrosis)	NA	0.004	NA
Cox modelling (tumour stage and presence of neutrophils)	NA	NA	0.0002

NA, not applicable; NS, not significant.

In FNA specimens, a high TIN score was strongly associated with a worse prognosis compared to a low TIN score (*p* < 0.0001). This observation retained significance in Cox proportional hazards regression analysis (*p* = 0.001). The presence of necrotic tissue was not assessed on the FNA samples. Cox modelling using stage and TINs was best at predicting OS from FNA samples (*p* = 0.0002). Two equivalent models were constructed using completed resections and FNA specimens; the associated *C*‐statistic of 0.804 computed between the two models lends support for the interchangeability of specimens at interpreting the presence of TINs and necrosis.

### TILs are not associated with outcome in ccRCC

Assessment of TILs in our resection specimen cohort showed that intratumoural or peritumour lymphocytes were present in most primary (76 and 100%, respectively) and metastatic (41 and 89%, respectively) samples. The presence of neither intratumoural nor peritumoural lymphocytes was significantly associated with outcome in the resection specimen cohorts. TILs were not assessed in the FNA samples.

### Neutrophils act directly on tumour tissue by releasing elastase, which successfully breaks down cell–cell adhesion

**Figure 3:**
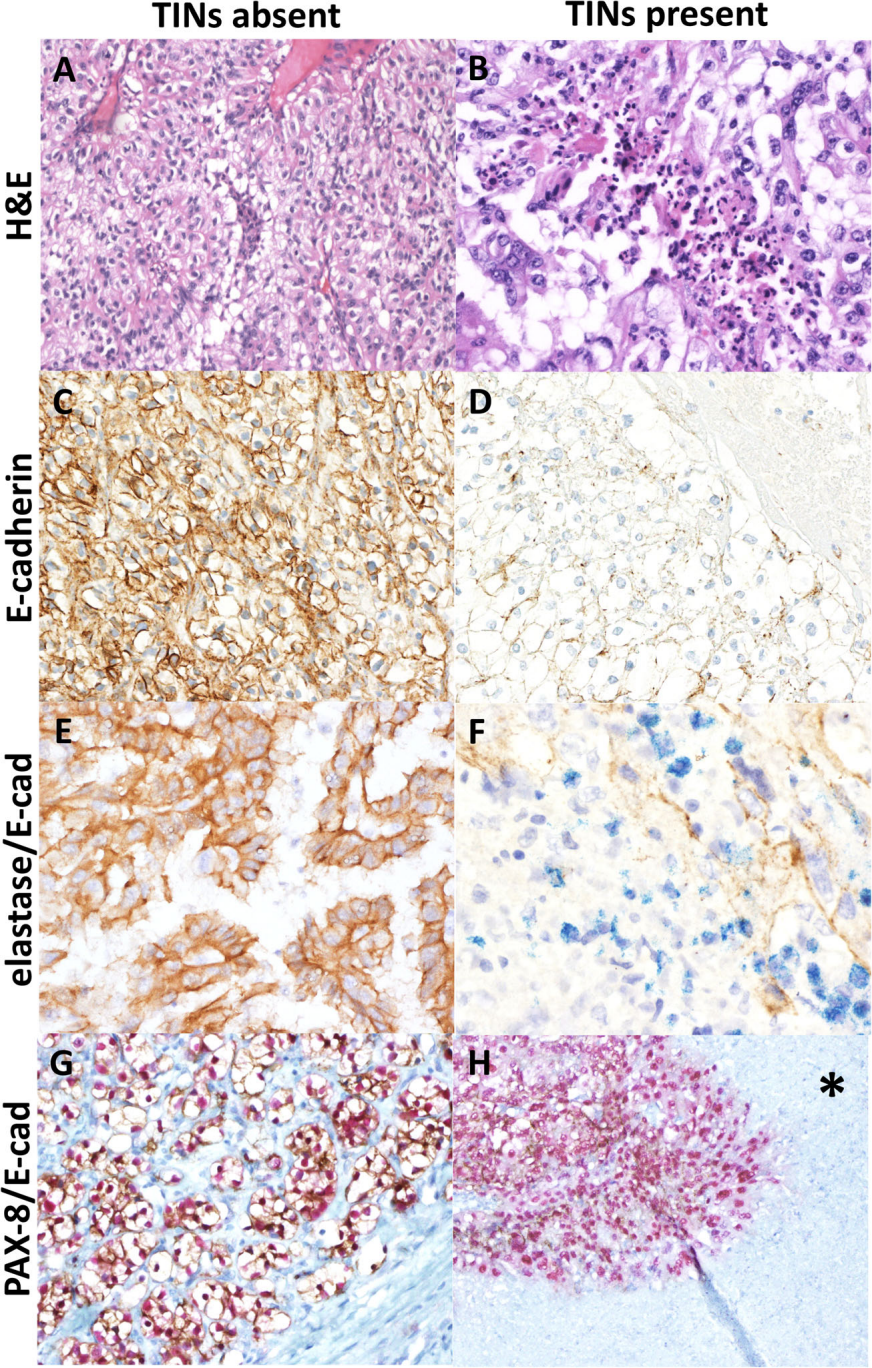
Staining characteristics of low and high TINs in ccRCC using (A and B) H&E, (C and D) E‐cadherin, (E and F) E‐cadherin (brown) and elastase (blue) dual stain, and (G and H) E‐cadherin (brown) and PAX‐8 (red) dual stain. *Area of necrotic tissue.

All ccRCCs with high TIN counts tested with E‐cadherin (*n* = 11) showed loss of expression, while among tested ccRCCs with low TIN counts (*n* = 5), E‐cadherin expression was always intact (Figure 3). We also performed IHC for Cit‐H3 (*n* = 14) to show that the neutrophils were positive for citrullinated H3, a marker of active NETosis. Cit‐H3 was positive in neutrophils and highlighted the presence of neutrophils in high TIN cases. Dual IHC for E‐cadherin/elastase showed that elastase was localised around areas with higher neutrophilic density and was also associated with loss of E‐cadherin expression. The dual PAX‐8 and E‐cadherin IHC confirmed that the loss of E‐cadherin was associated with the surrounding PAX‐8‐positive ccRCC tumour cells.

### CIBERSORT gene expression analysis shows that high neutrophil content in tumours is independently associated with worse prognosis

**Figure 4:**
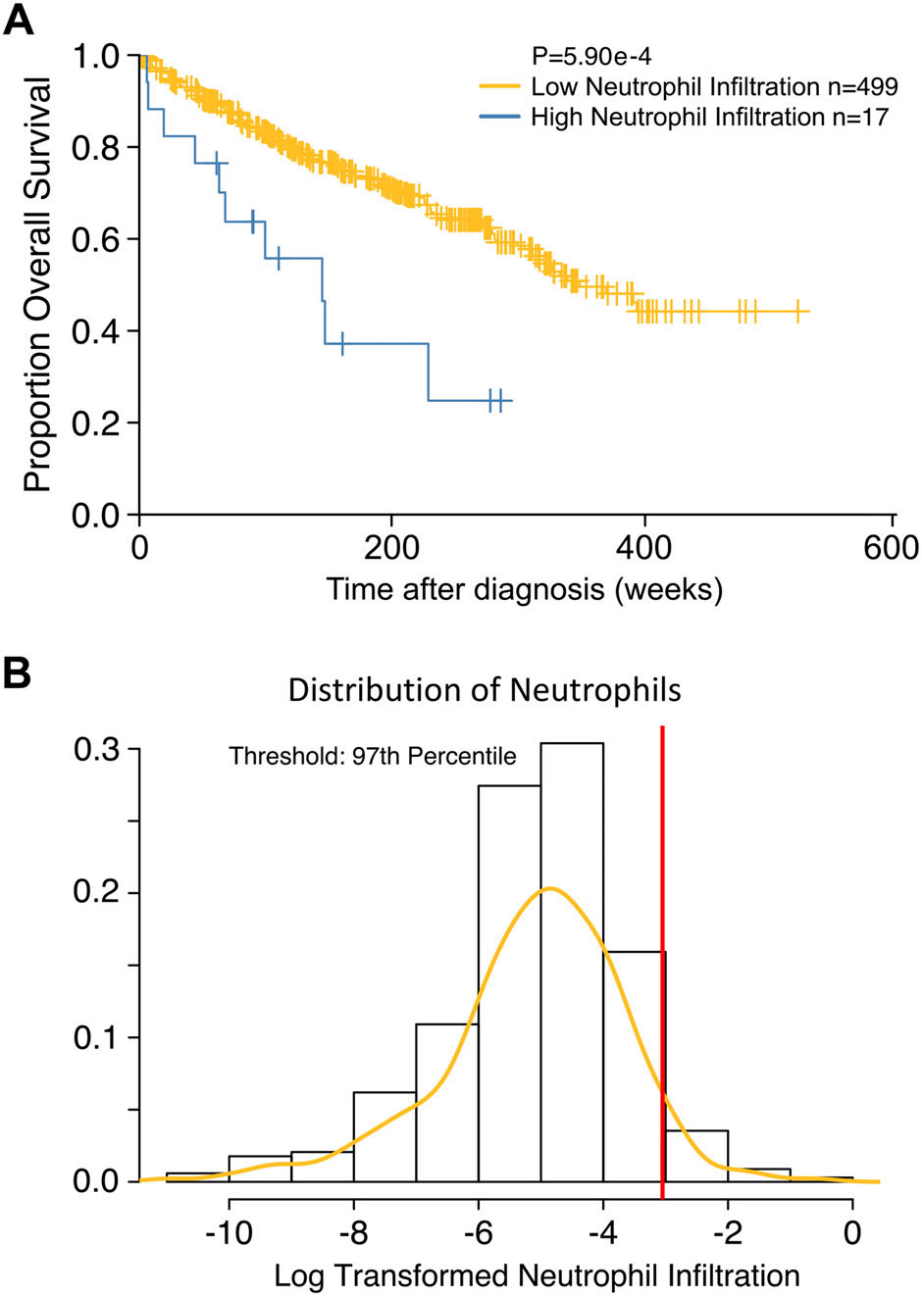
Kaplan–Meier analysis showing the association between increased neutrophilic markers (based on CIBERSORT) and (A) overall survival and (B) the distribution of the level of neutrophilic markers in the ccRCC TCGA cohort (*n* = 516).

The TCGA cohort used for this analysis included 516 patients and was previously described by The TCGA Research Network and Sato *et al* [25, 26]. The cohort included 65% men, and the median age was 61 years. The clinical stage was low (stage I–II) in 58% of cases, 61% of the cohort had low tumour stage (pT1–pT2), 13% had nodal metastasis at presentation, and 16% had distant metastases. The median follow‐up time was 1122 days. We stratified the ccRCCs into two groups based on their expression of neutrophilic markers at the 97th percentile using the CIBERSORT algorithm (Figure 4). Cases with low expression of neutrophilic markers had a median OS of 3.88 years, whereas those with high expression had a median OS of 2.18 years (*p* < 0.0001). Controlling for sex, age, and disease severity (using presence or absence of metastasis), we found that neutrophil infiltration continued to be significant (hazard ratio: 2.91, 95% CI: 1.39, *p* = 0.001). The multivariate regression model also showed that age (*p* < 0.0001) and presence of metastasis (*p* < 0.0001) were highly significant, but sex was not (*p* = 0.3).

## Discussion

Neutrophils have been associated with tumourigenesis for a long time, but the nature of this relationship is still poorly understood. We reviewed the morphology and molecular features of a series of ccRCC resections and FNA specimens in an attempt to shed light on the role of neutrophils in this disease. We also performed a gene expression‐based analysis to predict the neutrophilic infiltration in a publicly available database. We show that not only is the presence of neutrophils independently and strongly associated with a worse outcome, but TINs are also associated with the presence of elastase and the disruption of E‐cadherin cell‐cell adhesions.

The College of American Pathologists currently recommends including necrosis in ccRCC reports as it has been shown to be associated with worse prognosis [27, 28]. In our analysis, tumour necrosis showed a trend towards worse outcome, but it was not independently associated with prognosis. As controlling for TINs limited any association between necrosis and survival data, one might also suggest that the prognostic potential of tumour necrosis could be driven by its association with TINs. This observation supports the fact that TINs may be a superior prognostic marker to tumour necrosis in ccRCC. It is well known that cytokines released during tumour necrosis, but also by viable tumour cells in areas away from necrosis, can secrete chemokines to attract neutrophils [29, 30]. Furthermore, our CIBERSORT analysis of a large publicly available database showed a strong independent association between a predicted high number of infiltrating neutrophils and worse outcome.

Our results suggest that the location of TINs is important. While TINs around necrosis mostly aligned with size and the presence of necrosis, TINs away from the necrotic tissue remained statistically significant when controlling for these variables. This observation highlights the complexity of assessing the tumour microenvironment and support that there may be more than one mechanism for increased TINs. With a rapid increase in size, tumours often outgrow their blood supply and, through a cascade of events triggered by hypoxia, become necrotic [31, 32]. Necrotic tissue is known to attract immune cells, including neutrophils, through the release of inflammatory mediators such as IL‐1β, tumour necrosis factor α (TNFα), and IL‐8 [33, 34, 35]. It has been shown that certain tumours produce chemotactic agents, such as TNFα; IL‐17; CXCR2 ligands CXCL1, CXCL2, CXCL5, and CXCL6, to attract neutrophils [36, 37, 38, 39]. In turn, the neutrophils secrete other cytokines, metalloproteinases, and cytotoxic agents to the point where the immune reaction results in the damage of tumour cells [40]. In the latter scenario, as the immune cells are attracted by viable tumour cells, as opposed to necrotic ones, the presence of immune cells would be expected throughout the tumour cells. Furthermore, increasing evidence suggests that infiltrating neutrophils can releases elastase, which can either indirectly result in E‐cadherin downregulation in cancer cells or directly degrade its extracellular domain, resulting in tumour seeding [41, 42, 43].

It has been hypothesised that one of the mechanisms by which neutrophils promote invasion and dissemination is through the disruption of cell adhesion [15, 44]. In our series, the presence of activated neutrophils was always associated with loss of expression of E‐cadherin. Moreover, when vascular invasion was present, the tumour emboli were also mixed with neutrophils. One of the functions neutrophils use to fight infection, called NETs, involves the breakdown of its cell membrane and the release of DNA, histones, and granular constituents [45]. These NETs were recently shown to have an important role in protecting circulating tumour cells because of their DNA scaffold and fibrin net [46]. The released products have a role in helping to defend against microbial infections but also result in tissue injury, a prothrombotic environment, and angiogenesis [47, 48]. Together, these may promote tumour cell dissemination, as well as metastatic implantation and proliferation [49]. Recently, a study showed that citrullinated H3, which is a marker of activated neutrophils undergoing NETosis, was associated with worse prognosis and exacerbated the inflammatory response [50].

Non‐invasive tissue collection techniques such as FNA and other types of biopsy are popular in the diagnostic work‐up of ccRCC, but the amount of tissue is often limited. We show that TIN count in FNA specimens can predict survival similar to the count in larger resection specimens. This not only supports our hypothesis regarding the role of neutrophils in ccRCC, it also suggests that it is reproducible in smaller tissue samples. We did not identify any association between TILs and outcome. This is in line with previously published results, and although others have suggested that TILs are a marker of poor or good prognosis, their multivariate analyses either failed or were not reported [51, 52, 53, 54]. As our TIL analysis was limited to haematoxylin and eosin (H&E) scoring, we cannot exclude that the presence of certain lymphocyte subgroups, not distinguishable by H&E, could confer poorer prognosis, while others could predict a good outcome. More survival studies, accounting for important confounding variables, are needed to investigate the role of TILs in ccRCC.

Our study has several limitations. First, as histology allows us to assess only one plane, our criteria for counting neutrophils away from necrosis cannot take into account the possibility that necrosis could be closer on another plane. In addition, as neutrophils were studied *in vivo*, our approach did not allow us to confirm if the elastase was directly released by neutrophils actively undergoing NETosis or directly from the cytoplasmic granules of intact neutrophils. Studies on a larger cohort will be necessary to validate our findings and explore the role of the microenvironment in other renal cell carcinoma subtypes. As this cohort was enriched for primary ccRCCs associated with progression to metastatic disease, extrapolation of the TIN count to ccRCCs from the general population may not be reliable. An ongoing project is aimed at assessing the incidence of TINs in a larger non‐restricted cohort. Finally, although all primary resections and FNA samples were treatment naïve, complete treatment data were not available for the metastatic resection specimens and could not be accounted for during our survival analysis. However, all cases were managed with the same recommended treatment guidelines, and the use of adjuvant therapies is expected to be relatively homogeneous and its benefits limited at best; overall, this should have little impact on our survival analysis [55, 56].

In conclusion, we show that the presence of TINs, especially away from tumour necrosis, is a strong predictor of poor prognosis. We present a valid model of neutrophil‐mediated tumour dissemination whereby elastase released by activated neutrophils degrades E‐cadherins and allows tumour cell detachment. Not only do we suggest that TIN count be implemented in routine practice, but we provide evidence that it could be implemented in a variety of samples including FNAs. More work on TINs in ccRCC is needed to establish if it has a predictive role, especially in the context of the increased usage of immune checkpoint inhibitors.

## Authors contributions statement

BT‐C, JJL and DS designed the experiments. BT‐C, DDWT, MM, JK, NP, ZT, RLK and DS collected the data. BT‐C, RLK and DS reviewed the pathology. BT‐C, DDWT, H‐JEC and DS analysed the data. BT‐C and DS prepared the manuscript. BT‐C, DDWT, MM, JK, H‐JEC, RLK, JJL and DS reviewed the manuscript.
